# A Comprehensive Fault Diagnosis Method for Rolling Bearings Based on Refined Composite Multiscale Dispersion Entropy and Fast Ensemble Empirical Mode Decomposition

**DOI:** 10.3390/e21070680

**Published:** 2019-07-11

**Authors:** Weibo Zhang, Jianzhong Zhou

**Affiliations:** School of Hydropower & Information Engineering, Huazhong University of Science and Technology, Wuhan 430074, China

**Keywords:** fault diagnosis, rolling bearing, fast ensemble empirical mode decomposition, refined composite multiscale dispersion entropy, max-relevance and min-redundancy, random forest

## Abstract

This study presents a comprehensive fault diagnosis method for rolling bearings. The method includes two parts: the fault detection and the fault classification. In the stage of fault detection, a threshold based on refined composite multiscale dispersion entropy (RCMDE) at a local maximum scale is defined to judge the health state of rolling bearings. If the bearing is in fault, a generalized multi-scale feature extraction method is developed to fully extract fault information by combining fast ensemble empirical mode decomposition (FEEMD) and RCMDE. Firstly, the fault vibration signals are decomposed into a set of intrinsic mode functions (IMFs) by FEEMD. Secondly, the RCMDE value of multiple IMFs is calculated to generate a candidate feature pool. Then, the maximum-relevance and minimum-redundancy (mRMR) approach is employed to select the sensitive features from the candidate feature pool to construct the final feature vectors, and the final feature vectors are fed into random forest (RF) classifier to identify different fault working conditions. Finally, experiments and comparative research are carried out to verify the performance of the proposed method. The results show that the proposed method can detect faults effectively. Meanwhile, it has a more robust and excellent ability to identify different fault types and severity compared with other conventional approaches.

## 1. Introduction

Rotating machinery is a major part of mechanical equipment, including many engineering fields such as power, chemical, metallurgy, and machinery manufacturing [1,2,3,4]. Its working condition directly affects the safety and stability of mechanical operation. Bearings are one of the most common and fragile general parts in rotating machinery, and their health is directly related to whether the machine can operate normally. Therefore, it is necessary to execute health monitoring and fault diagnosis of the bearing, and it has drawn considerable attention and research.

As bearings operate, they unavoidably suffer from cracks, corrosion, spalling and other factors, which cause the vibration signals to exhibit nonlinear dynamic characteristic. Therefore, how to effectively extract and detect fault characteristics of bearings is crucial in fault diagnosis [5]. Data-driven based methods are the mainstream methods for solving various problems [6,7], including statistical methods, signal processing methods, and artificial intelligence-based methods. For instance, Santos [8] employed a data-driven system based on dynamic principle components to detect faults. However, it cannot extract non-linear characteristics. Recently, with the development of nonlinear dynamic technologies, nonlinear dynamic methods have been widely applied in many areas, such as biomedicine and image processing [9,10]. Due to their extraordinary advantages in extracting nonlinear characteristics of vibration signals, many entropy-based methods, such as approximate entropy [11], sample entropy [12], and permutation entropy (PE) [13,14], are widely applied for fault diagnosis. However, the quality of the approximate entropy result depends on the length of data. It will result in bad estimate values when analyzing short datasets. Sample entropy has addressed the shortcoming of approximate entropy, but it brings a high computational complexity. Different from approximate entropy and sample entropy, PE has a simple concept and fast computing speed. However, it drops some information of amplitudes while mainly concerning the order of amplitude values. Therefore, a recent entropy-based method called dispersion entropy (DE) was introduced by Azami [15] to overcome these shortcomings, and it has been adopted to detect bearing faults due to its excellent performance in measuring the complexity and randomness of vibration signals [16]. 

However, the above entropy-based methods extract fault characteristics only at a single scale. It does not always get the desired results as it will lose some important information. To overcome the drawback, multi-scale sample entropy (MSE) and multi-scale permutation entropy (MPE) were proposed by Costa [17,18] and Aziz [19], respectively. They can analyze vibration signals over multiple scales by introducing a coarse-graining time series. Accordingly, multi-scale dispersion entropy (MDE) and refined composite MDE (RCMDE) were further developed by Azami [20] to overcome the shortcomings of MSE and MPE. Compared with single-based entropy methods, multi-scale entropy-based methods have greatly improved the performance and achieved growth applications in fault diagnosis [21,22,23]. Therefore, RCMDE is exploited to measure the complexity and randomness of vibration signals in this study, and it will be considered as a tool to detect the health of bearings in the study. 

Nevertheless, RCMDE directly acts on the original signal, which cannot reveal the inherent characteristic of vibration signals. The diagnosis results may be not ideal when the bearing fault is of different type and severity. Considering the nonlinear and non-stationary characteristics of vibration signals, entropy-based methods are often combined with the time–frequency analysis technique to extract the fault information from bearings [24]. The typical time–frequency analysis technique, such as wavelet transform (WT) [25], variational mode decomposition (VMD) [26], empirical mode decomposition (EMD) [27] and local mean decomposition (LMD) [28,29], is firstly to decompose complicated signals into a set of components which represent its intrinsic characteristic. Then, the entropy-based approach is adopted to measure the complexity of the components. For instance, Li [30] combined LMD and MPE to extract features from rolling bearings. Zhang [31] employed ensemble empirical mode decomposition (EEMD) and PE to identify fault type and severity. Tan [32] applied VMD and fuzzy entropy to construct fault feature vectors, but they extracted fault information only according to a single component or single scale [30,31,32,33]. The potential fault information hidden in other scales or other components also play important roles for fault diagnosis. Thus, we present a generalized multi-scale feature extraction method to extract fault features from different components as well as multiple scales. EMD is one of most popular and widely used time–frequency tools, but mode mixing problems exist. EEMD is an advanced version of EMD that mitigates the mode-mixing problem of EMD. However, its computational cost is too high when the number of samples is large. Fast EEMD (FEEMD), presented by Wang and Yeh [34], is an effective time–frequency tool. Compared with EEMD, it is considerably faster while both decomposition results are nearby and reliable [35,36,37]. Therefore, FEEMD is adopted to decompose original vibration signals into multiple components (IMFs). Then, RCMDE values of multiple IMFs are calculated to formulate candidate feature pools. To reduce the computational burden and increase classification accuracy, minimum-redundancy maximum-relevancy (mRMR) [38] is employed to select sensitive features to generate the final feature vector. Then, the random forest (RF) classifier [39,40,41,42] is adopted to train and test the final feature vectors to identify different fault working conditions.

The contribution of this work is the development of a multi-step comprehensive fault diagnosis method including fault detection and fault classification. The preliminary fault detection is based on statistical analysis of RCMDE, which is fast and can be easily applied to judge the health state of bearings. If the bearing is detected to be healthy, the program outputs “Normal” and is terminated. Otherwise, the proposed generalized multi-scale feature extraction method based on FEEMD and RCMDE is employed for fault diagnosis, which can fully extract fault information. Subsequently, to further improve the efficiency and diagnostic accuracy, the mRMR is adapted for feature selection and the RF classifier for fault classification. The proposed fault diagnosis model is applied to the vibration signals of bearings, and the diagnosis results show its robust diagnosis performance for different types and severities of faults. Moreover, compared with the conventional single-step diagnosis approach, this stepwise diagnostic strategy is more in line with practical engineering applications.

The rest of this paper is organized as follows: Section 2 presents the basic theory of FEEMD, DE, RCMDE, and mRMR. Section 3 gives details of the proposed method. Section 4 provides the analysis and results of the experiments. Finally, the conclusion is reported in Section 5.

## 2. Basic Theory

### 2.1. Fast Ensemble Empirical Mode Decomposition (FEEMD)

FEEMD, developed by Wang [34], is an improved version of EEMD, which can decompose vibration signals into a series of IMFs and a residue according its inherent structure. It not only speeds up the computational speed of EEMD, but also achieves the same good decomposition results as EEMD, which has been verified in the literature [36,37]. The accelerated calculation of FEEMD is implemented by optimizing the program of EMD. Similar to EEMD, the procedure of FEEMD can be briefly described as follows:

(1) Add a white noise series $n_{j}(t)$ to the original signal s(t) (repeated M realizations),

(1) $$s_{j}\left(t\right)=s(t)+n_{j}(t),$$

where $n_{j}(t)$ represents the added noise in the *j-*th realization.

(2) Decompose the noise-added signal $s_{j}\left(t\right)$ into *n* IMFs and a residue using EMD,

(2) $$s_{j}(t)=\sum_{i=1}^{n}c_{ij}(t)+r_{j}(t),$$

where $c_{ij}(t)$ and $r_{j}(t)$ represent the *i-*th IMF and residue in the *j-*th realization, respectively. 

(3) Compute the ensemble mean as follows:

(3) $$c_{i}(t)=\frac{1}{M}\sum_{j=1}^{M}c_{ij}(t),$$

(4) $$r_{}(t)=\frac{1}{M}\sum_{j=1}^{M}r_{j}(t),$$

where $c_{i}(t)$ represents the *i-*th IMF of FEEMD, and r(t) represents the residue of FEEMD.

### 2.2. Dispersion Entropy and Refined Composite Multiscale Dispersion Entropy 

#### 2.2.1. Dispersion Entropy

For a given time series: *x* = *x_1_*, *x_2_*, …, *x_N_*, the dispersion entropy (DE) can be described as follows [15]:

(1) Firstly, *x_j_* (*j* = 1,2, …, *N*) are mapped into *y* = {*y_1_*, *y_2_*, …, *y_N_*} from 0 to 1 by a normal cumulative distribution function (NCDF), and each *y_j_* are assigned to an integer from 1 to *c* by linear algorithms. The mapped signal can be denoted as follows:

(5) $$z_{j}^{c}=\mathrm{round}(c.y_{j}+0.5),$$

where $z_{j}^{c}$ denotes the *j*-th member of the classified time series. 

(2) Define the embedding vector $z_{j}^{m,c}$ with embedding dimension *m* and time delay *d* following the equation:

(6) $$z_{i}^{m,c}=\{z_{i}^{c},z_{i+d}^{c},\ldots ,z_{i+(m-1)d}^{c}\},$$

where *i* = 1,2, …, *N* − (*m* − 1)*d*, each time series $z_{i}^{m,c}$ is mapped to a dispersion pattern, and $z_{i}^{c}=v_{0},z_{i+d}^{c}=v_{1},\ldots ,z_{i+(m-1)d}^{c}=v_{m-1}$.

(3) The relative frequency of each potential dispersion patterns can be given by:

(7) $$p(\pi_{v_{0}}{}_{v_{1}}\ldots_{v_{m-1}})=\frac{\mathrm{Number}\{i|i\leq N-(m-1)d,\;z_{i}^{m,c}\mathrm{has}\;\mathrm{type}\;\pi_{v_{0}}{}_{v_{1}}\ldots_{v_{m-1}}\}}{N-(m-1)d},$$

(4) Finally, the DE can be computed as follows:

(8) $$\mathrm{DE}(x,m,c,d)=-\sum_{\pi =1}^{c^{m}}p(\pi_{v_{0}}{}_{v_{1}}\ldots_{v_{m-1}})\cdot \ln(p(\pi_{v_{0}}{}_{v_{1}}\ldots_{v_{m-1}})),$$

where *m* is the embedding dimension and *d* is the time delay.

#### 2.2.2. Refined Composite Multiscale Dispersion Entropy (RCMDE)

RCMDE, developed by Azami in 2017 [20], is an improved method based on DE. The RCMDE value is calculated as the Shannon entropy of the coarse-graining time series. The progress of RCMDE is as follows:

(1) Construct multiple coarse-graining series:

(9) $$x_{k,j}^{\tau }=\frac{1}{\tau }\sum_{i=k+(j-1)\tau }^{k+j\tau -1}x_{i},\;1\leq j\leq \frac{N}{\tau },1\leq k\leq \tau ,$$

where *τ* is the scale factor and $x_{k,j}^{\tau }$ denotes the *k*-th coarse-grained time series of *x.*

(2) For each scale factor, RCMDE is defined as follows:

(10) $$\mathrm{RCMDE}(x,m,c,d,\tau )=-\sum_{\pi =1}^{c^{m}}\bar{p}(\pi_{v_{0}}{}_{v_{1}}\ldots_{v_{m-1}})\cdot \ln(\bar{p}(\pi_{v_{0}}{}_{v_{1}}\ldots_{v_{m-1}})),$$

where $\bar{p}(\pi_{v_{0}}{}_{v_{1}}\ldots_{v_{m-1}})=\frac{1}{\tau }\sum_{1}^{\tau }p_{k}^{\left(\tau \right)}$ with the relative frequency of the dispersion pattern π in $x_{k}^{\left(\tau \right)}(1\leq k\leq \tau )$.

#### 2.2.3. Parameter Settings of RCMDE

In RCMDE, four essential parameters need to be set: embedding dimension *m*, the number of classes *c*, time delay *d* and scale factor *τ*. For embedding dimension *m*, if it is too small, the dynamic change of the signal is hard to detect. If it is too large, small variations are not noticed. For the number of classes *c*, it ought to be larger than one to avoid only one dispersion pattern existing. If it is too small, distant amplitude values may be classified into the same class. If it is too large, their class may be changed due to a small difference, and the DE method is easily disturbed by noise. It is generally set from 4–8. Moreover, if *c* or *m* is too large, the calculation cost will increase, and the result will be more trustworthy. *c^m^* must be smaller than the length of the signal. For time delay *d*, it is suggested that *d* = 1. For scale factor *τ*, if it is too small, the fault information cannot be extracted effectively. If it is too large, the computational cost will increase. It is suggested that the maximum scale factor *τ*_max_ = 20. In this study, we set *m* = 4, *c* = 6, *d* = 1 and *τ*_max_ = 20 according to the literature [15,16,20].

### 2.3. Max-Relevance And Min-Redundancy (mRMR) 

In pattern recognition, the computational cost will be large and the classification accuracy will also be reduced if the number of features is too large. The mRMR is proposed by Peng et al. [38] to select superior features based on mutual information. The basic idea of mRMR is as follows:

Given two random variables *x* and *y*, their similarity can be measured by mutual information I(X;Y):

(11) $$I(X;Y)=\iint p(x,y)\log\frac{p(x,y)}{p(x)p(y)}dxdy,$$

where *p*(*x*), *p*(*y*), *p* (*x*, *y*) denotes the probability of *X*, probability of *Y*, and probabilistic density functions of *X* and *Y*, respectively.

Feature selection aims to seek a feature set *S* with features *X*, which has large mutual information on the target class C based on max-relevance criterion:

(12) $$\max D(S,c),\;D=\frac{1}{\left|S\right|}\sum_{x_{i}\in S}I(x_{i};c),$$

where |*S*| is the number of features in subset *S.*

To avoid redundancy in selected features, the min-redundancy is to eliminate the features which have a large dependency and minimal redundancy. The min-redundancy is defined as follows:

(13) $$\min R,\;R=\frac{1}{{\left|S\right|}^{2}}\sum_{x_{i},x_{j}\in S}I(x_{i};x_{j}),$$

Thus, the mRMR is based on both the criterion of max-relevance criterion and min-redundancy. The operator can be optimized as:

(14) maxϕ(D,R), ϕ=D−R,

(15) maxϕ(D,R), ϕ=D/R,

Suppose we have found a feature set *S_m_*_−1_ with *m* − 1 features, and the mRMR is seeking the *m-*th feature from feature set {*X* − *S_m_*_−1_}. Using an incremental search method_._ it should meet the following equations:

(16) $$\underset{x_{j}\in X-S_{m-1}}{\max}[I(x_{j};c)-\frac{1}{m-1}\sum_{x_{i}\in S_{m-1}}I(x_{j};x_{i})],$$

(17) $$\underset{x_{j}\in X-S_{m-1}}{\max}[I(x_{j};c)/\frac{1}{m-1}\sum_{x_{i}\in S_{m-1}}I(x_{j};x_{i})],$$

## 3. The Proposed Method

In this study, based on advantages of FEEMD, RCMDE, mRMR, and RF, a comprehensive fault diagnosis method is proposed for fault diagnosis of rolling bearings. The method includes fault detection and fault classification.

### 3.1. Fault Detection

DE is able to measure the complexity and randomness of signals. Similar to PE and approximate entropy, DE has the capability of detecting faults [16]. However, when many types of faults exist, the DE values between normal and certain fault working conditions are very close. It cannot effectively distinguish between the normal and all fault working conditions.

As with the increase of scale factor, the RCMDE values of normal vibration signals change slowly, while RCMDE values of fault vibration signals rapidly decrease. When the scale factor is large enough, the differences of RCMDE values between normal and fault workings will be very apparent. Thus, based on different sensitivities to the scale factor, RCMDE values at a large scale can be employed to distinguish between normal and fault working conditions. To achieve a better discrimination effect, a threshold is defined based on RCMDE values at a local maximum scale factor *τ*_max_ = 20 to detect faults in this study.

### 3.2. Fault Classification

If the bearing is detected to be faulty, the faulty vibration signals will be further analyzed to classify all fault working conditions. In order to fully extract the fault information of bearings, we presented a generalized multi-scale feature extraction method based on FEEMD and RCMDE. Different from traditional multi-scale methods that extract nonlinear features either by calculating multi-scale entropy of a single component [30,33], or single entropy of multiple components [31,43], we extracted fault features via different components as well as multiple scales. The basic idea is to decompose the fault vibration signals into multiple IMFs that represent its inherent oscillations, then RCMDE was applied to extract the fault characteristic of the IMFs. Thus, a candidate feature pool is formed by these multi-scale features, which can completely extract non-linear fault characteristics. Next, the mRMR is employed to select superior and sensitive features from the candidate feature pool. Finally, the RF classifier was used to classify different fault working conditions.

**Figure 1:**
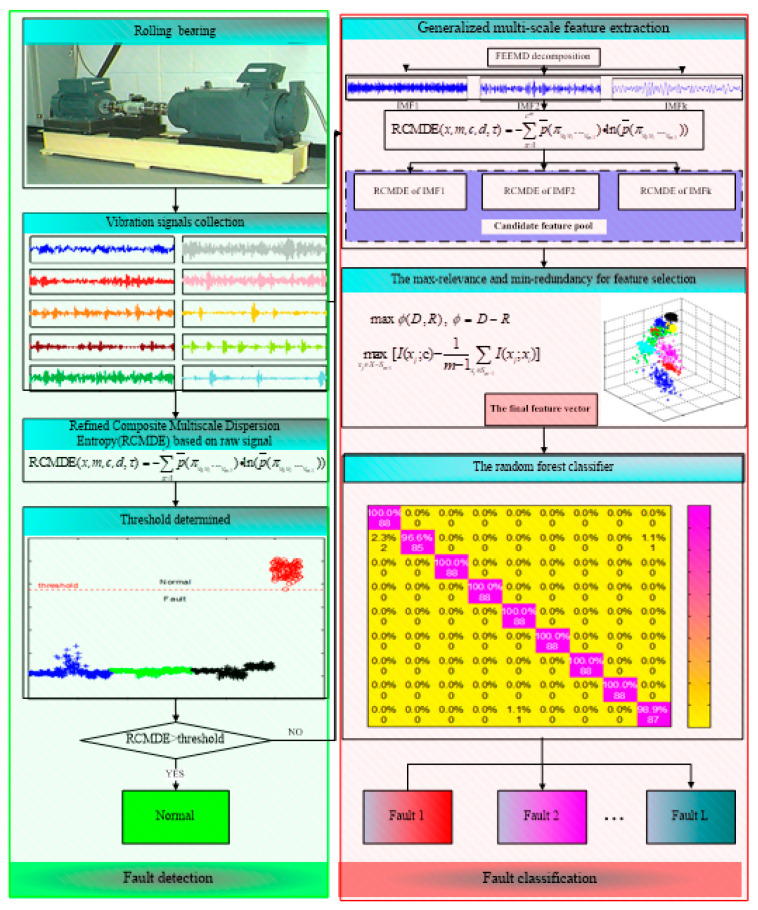
The flowchart of proposed method.

The flowchart of the proposed method is presented in Figure 1, and the general steps of the ensemble fault diagnosis method are presented as follows:

(1) Collect the vibration signals under different working conditions of rolling bearings.
(2) Divide the vibration signals into non-overlapped samples.
(3) Calculate the RCMDE values of vibration signals at different scale factors. Find out a threshold based on RCMDE to judge the health status of a bearing. If it is healthy, output normal to present the working condition of the bearing. Otherwise, identify the faults of different types and severity in the next steps.
(4) The fault vibration signals are decomposed into multiple IMFs by FEEMD.
(5) The RCMDE values of the first several IMFs are calculated to construct the candidate feature pool.
(6) The mRMR is employed to select the sensitive features from the candidate feature pool to generate the final feature vectors.
(7) The final feature vectors are fed into the random forest classifier to identify different fault types and severity.

The pseudocode of the proposed fault diagnosis algorithm (see Algorithm 1) is presented as follows:
**Algorithm 1. The Pseudocode of the Fault Diagnosis Algorithm**1 Input the vibration signals of *N* different working conditions 2 Calculate the RCMDE values *R_i_* of different working conditions at scale factor *τ*_max_ 3 Define a threshold *§* 4 If *R_i_* > *§* 5 Output “Normal” 6 Else 7 Decompose the fault vibration signals of *L* different fault working conditions into *m* IMFs 8 Calculate the RCMDE values of the first *k* IMFs at scale factor *τ*, (*τ* = 1,2, …, *τ*_max_) 9 Then, for fault working conditions, the candidate feature pool is formed with a size of *E* × *F*, (*E* is number of fault sample, *F* = *k* × *τ*_max_) 10 For training samples *E_train_* × *F*, training label *L_train_*, select *s* features from ranked features by mRMR, obtain *E_train_* × *S_train_* 11 For testing samples *E_test_* × *F*, select *s* features according to ranking results of training samples, obtain *E_test_* × *S_test_* 12 Put *E_train_* × *S_train_*, *L_train_* and *E_test_* × *S_test_* into RF classifier 13 Obtain test label *L_test_* 14 Output fault working condition

## 4. Experiment Results

### 4.1. Experimental Data 

To research the potential application of the proposed method, experimental data of bearings provided by Case Western Reserve University [44] were used in this study. The motor operated under 0 horsepower with a speed of 1797 rpm. The vibration data was collected from drive end bearings at a sampling frequency of 12,000 Hz. It mainly contains one normal and nine fault working conditions. The fault working conditions include three fault types: ball fault, inner raceway fault and outer raceway fault (located at three o’clock). Each fault types have different severities with fault diameters of 0.001 inches, 0.014 inches and 0.021 inches. In the study, the vibration data of each working condition was divided into 110 non-overlapped samples, and each sample consisted of 1024 data points. To agree with the actual engineering application, 20 percent of each working condition sample was randomly selected for training, and the remains were used as test samples to validate the effectiveness of the presented method. The experiments were performed in MATLAB 2013a and tested on a computer with Intel Core 2.6 GHz central processing unit (CPU) and 4.0 GB random access memory (RAM). The details of the experimental data are presented in Table 1.

### 4.2. Result and Analysis

**Figure 2:**
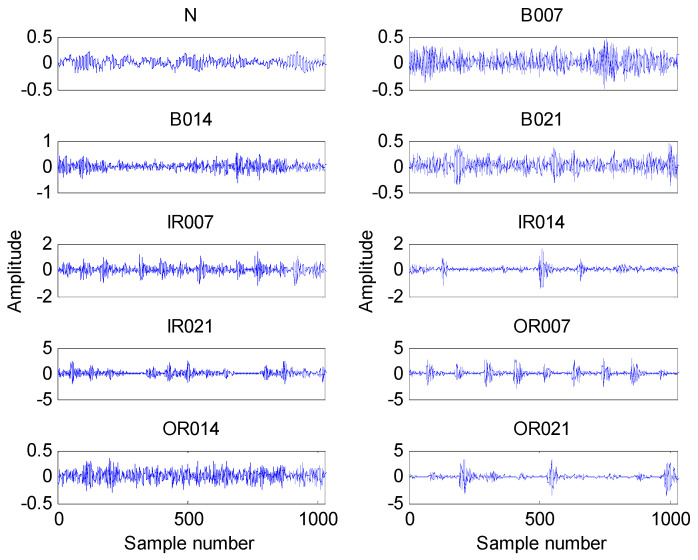
The waveform of all 10 working conditions.

**Figure 3:**
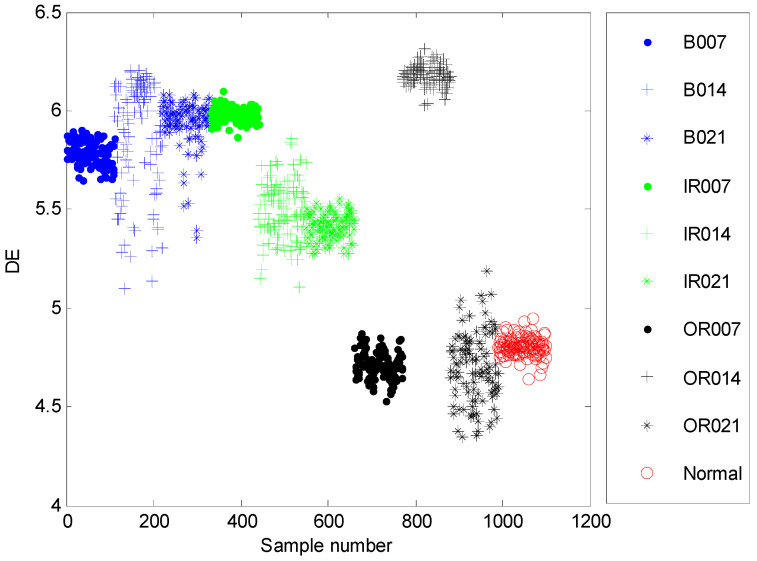
The dispersion entropy (DE) distribution of all samples.

**Figure 4:**
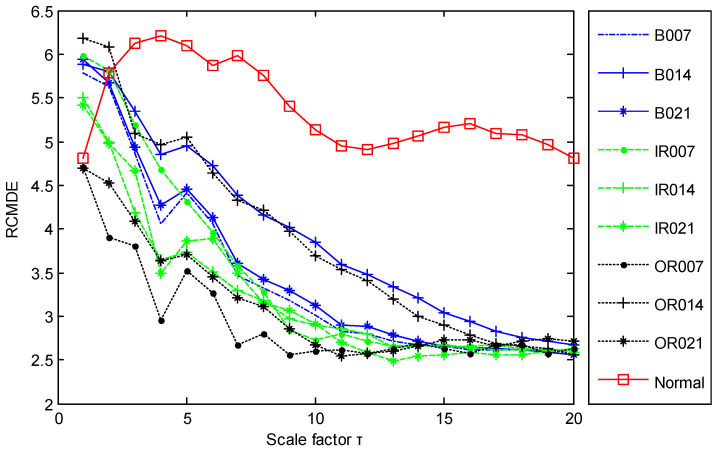
The RCMDE values of different scales for all 10 working conditions.

**Figure 5:**
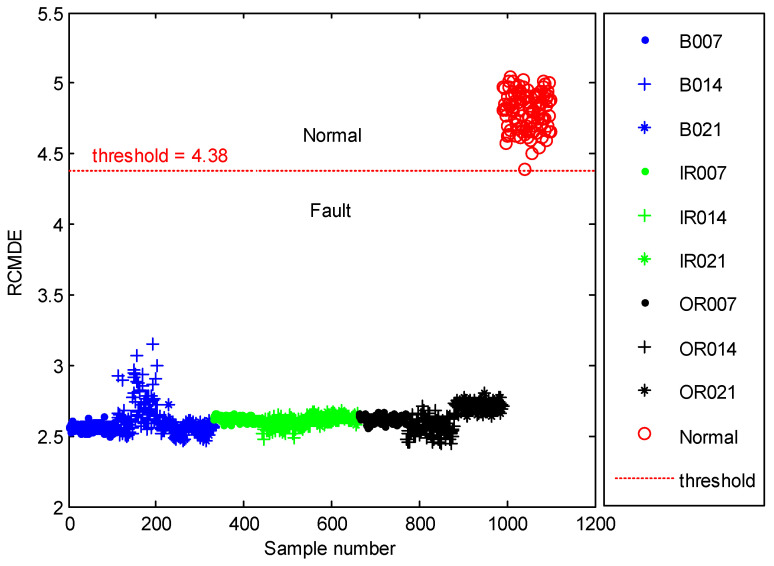
The RCMDE distribution of all samples at scale factor *τ* = 20.

The time domain waveforms of all 10 work conditions of bearings are displayed in Figure 2. It is difficult to identify different working conditions according their raw vibration signals. In the previous study, the single scale entropy value is usually considered as a measure to detect faults of bearings (such as PE value in [31,42]). Figure 3 presents the DE values of all samples. As Figure 3 shows, the DE values cannot effectively distinguish between normal and fault working conditions. The DE values of normal working conditions is very close to that of OR007 and OR021. Figure 4 shows the average RCMDE values of the original sample data as a function of the scale factor for all 10 working conditions. It appears that the interval of the RCMDE values between normal and fault conditions gradually became larger as the scale factor increases. When the scale factor is larger than four, the RCMDE values of normal and fault working conditions show differences, which can be used to detect the health status of bearings. When the scale factor reaches 20, the RCMDE values of different faults remain consistent and reach lower values. At this time, the RCMDE value of normal working conditions remains high, which is significantly different from fault working conditions. Thus, RCMDE values at scale factor 20 were considered and selected as a measure to detect faults of bearings. The RCMDE distribution of all samples at scale factor *τ =* 20 is displayed in Figure 5. From Figure 5, the RCMDE value of normal conditions was significantly higher than that of fault conditions. The threshold value (4.38) at the red dotted line can clearly distinguish the normal and fault working conditions. To evaluate the effect of the proposed method in the stage of fault detection, the indicator of fault detection rate (FDR) and false alarm rate (FAR) is adopted in this paper. From Figure 5, all normal samples are distributed above the threshold line, while all fault samples are below the threshold line. From the results of statistical analysis, the indicator of FDR achieves 100% and the FAR is 0%. The larger the FDR and the smaller the FAR, the better the performance. Obviously, the proposed method has an excellent performance in detecting faults.

**Figure 6:**
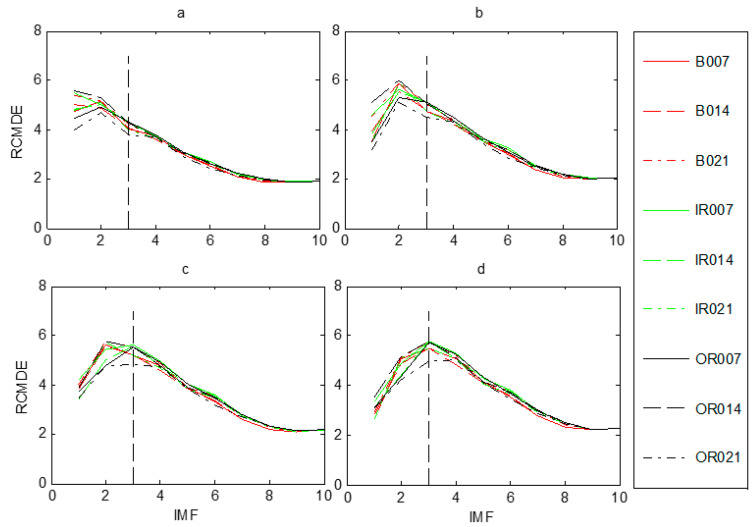
IMF-RCMDE of different fault working conditions at four scale factors *τ*: (**a**) *τ =* 1, (**b**) *τ = 2* (**c**) *τ =* 3, (**d**) *τ =* 4.

Judging the health state of the rolling bearing is the first step in fault diagnosis. Once the bearings were in fault, the proposed fault diagnosis model was utilized to discriminate different fault types and fault severities. According to the flowchart of the presented approach, displayed in Figure 1, the raw vibration signals were firstly decomposed into a set of IMFs by FEEMD. In FEEMD, the ensemble number *M* = 100, and the standard deviation of added white noise *sd* = 0.2 Then, the RCMDE was employed to measure the complexity of each IMF. Figure 6 presents RCMDE values as a function of IMF for all fault working conditions at four different scale factors. As Figure 6 shows, the RCMDE values of different fault working conditions are distinct at each of the IMFs, which can be applied to identify faults of different types and severities. The RCMDE values of the first several IMFs are higher than that of latter IMFs. The reason for this is that when the bearing is in fault, the fault information is mainly reflected in the high frequency components (that is, the first several IMFs). Meanwhile, from Figure 6a–d, it appears the RCMDE values of the first three IMFs show more differences between fault conditions, whereas the latter IMFs are quite consistent in RCMDE values. Thus, the first three IMFs of each samples are selected to calculate the RCMDE values as they contributed a lot to fault classification. Moreover, from Figure 4 and Figure 6, the fault information hidden at different scale factors also plays an important role for classification, which will improve the accuracy and reliability of fault classification. In the study, we set maximum scale factor *τ*_max_ *=* 20. For all fault samples, we can obtain a candidate feature pool with a size of 990 × 60. However, the preliminary feature vector formed by the candidate feature pool is high-dimensional, which will enlarge the computational cost and reduce the classification accuracy. Hence, the mRMR technique was used to extract 12 sensitive features to generate the final feature vectors. Finally, the selected sensitive feature vectors were presented into a multi-classifier RF for fault classification.

**Figure 7:**
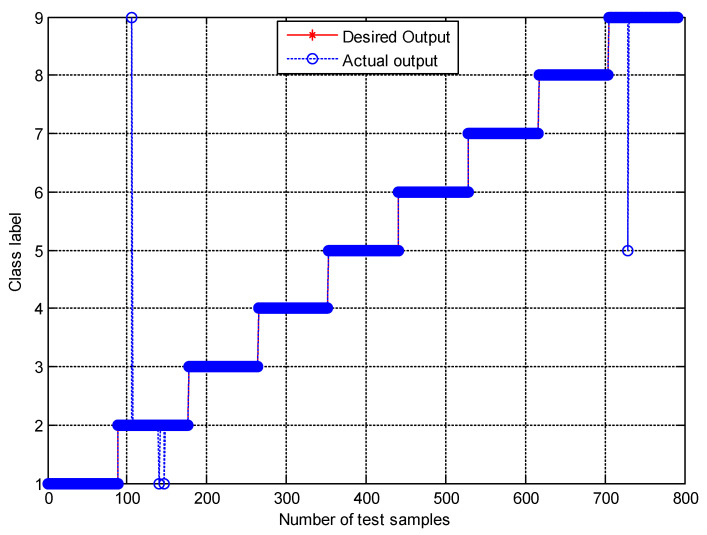
The classification results by the presented method.

**Figure 8:**
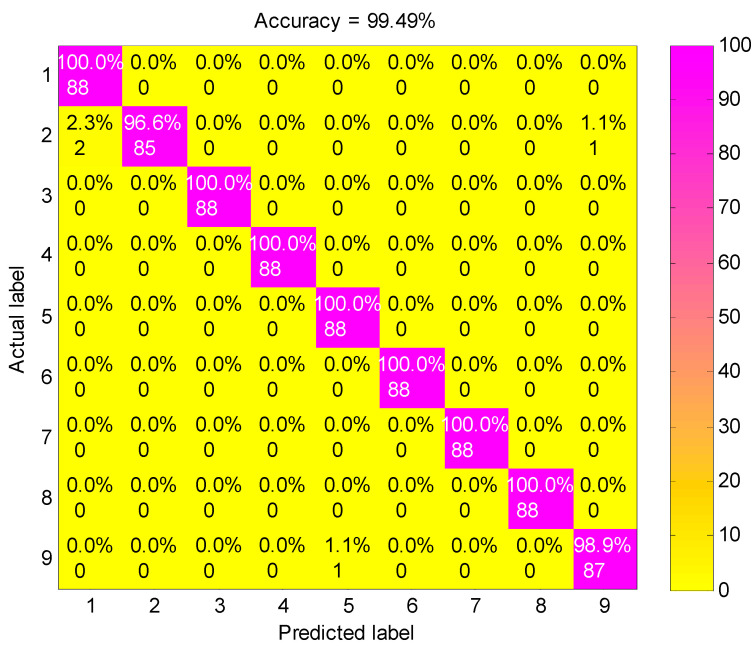
The confusion matrix of the presented method.

Figure 7 shows the classification results of nine fault working conditions for one trial by the presented method. The experimental analysis is a nine-level classification problem. Among the 792 test samples, one B014 (label 2) sample was misclassified into OR021 (label 9), two B014 samples were misclassified into B007 (label 1), and one OR021 sample was misclassified into IR014 (label 5). The confusion matrix is a standard format for accuracy evaluation, which can reflect the details of predicted results and actual results in model assessment. Figure 8 shows the confusion matrix of the presented method. It is obvious that the accuracy of the second fault working condition (B014) and the ninth fault working condition (OR021) is 96.6% and 98.9%, respectively, and the accuracies of the other fault working conditions achieves 100%. The classification accuracy of the proposed method for all fault working conditions reaches as high as 99.49%, showing a robust recognition ability for faults of different categories and severities. 

**Figure 9:**
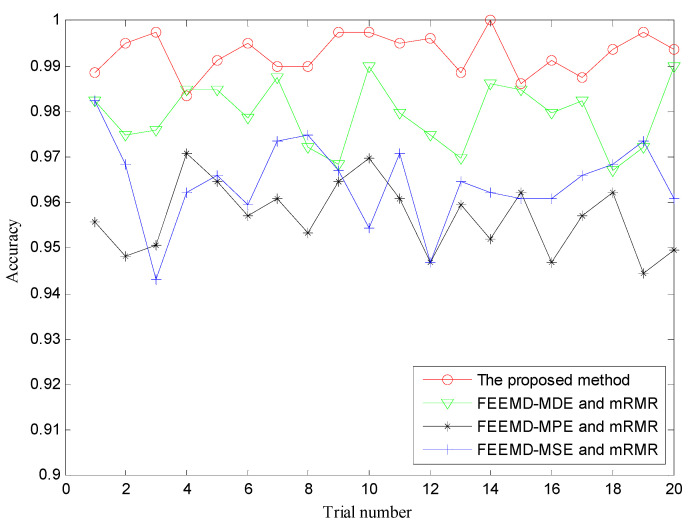
The diagnosis results of four methods for 20 trials. MDE = multi-scale dispersion entropy; MSE = multi-scale sample entropy; MPE = multi-scale permutation entropy.

In order to prove the superiority of the proposed method, several other typical multi-scale entropy-based methods such as MDE, MPE and MSE were also performed to identify different fault types and severities. To remain consistent with the proposed method, similarly, FEEMD was firstly used to decompose the raw vibration signals into multiple IMFs, and the above three multi-scale entropy-based methods were employed to measure the complexity of raw vibration signals. Then, the important features were selected by mRMR and presented into RF for fault identification. For FEEMD-MDE, parameters were set as follows: *M =* 100, *sd* = 0.2, *m* = 4, *c* = 6, *t* = 1, *τ*_max_ = 20. For FEEMD-MPE, *M* = 100, *sd* = 0.2, *m* = 6, *t* = 1, *τ*_max_ = 20. For FEEMD-MSE, *M* = 100, *sd* = 0.2, *m* = 2, *t* = 1, *rd* = 0.15, *τ*_max_ = 20. Here, *M* is the ensemble number of FEEMD, *sd* is the standard deviation of added white noise in FEEMD, *m* is the embedding dimension, *c* is the number of classes, *τ* is time delay, *rd* is the tolerance of the signal and *τ*_max_ is the largest scale factor. To reduce the impact of randomness, the trial of each method was repeated 20 times. The classification accuracies of different methods are shown in Figure 9 and Table 2. It is obvious that the accuracy of the presented method is better than that of the other three methods. Meanwhile, the highest classification accuracy of the proposed method reaches 100%, and the average accuracy is also higher than the others, showing its robust ability for fault classification.

**Figure 10:**
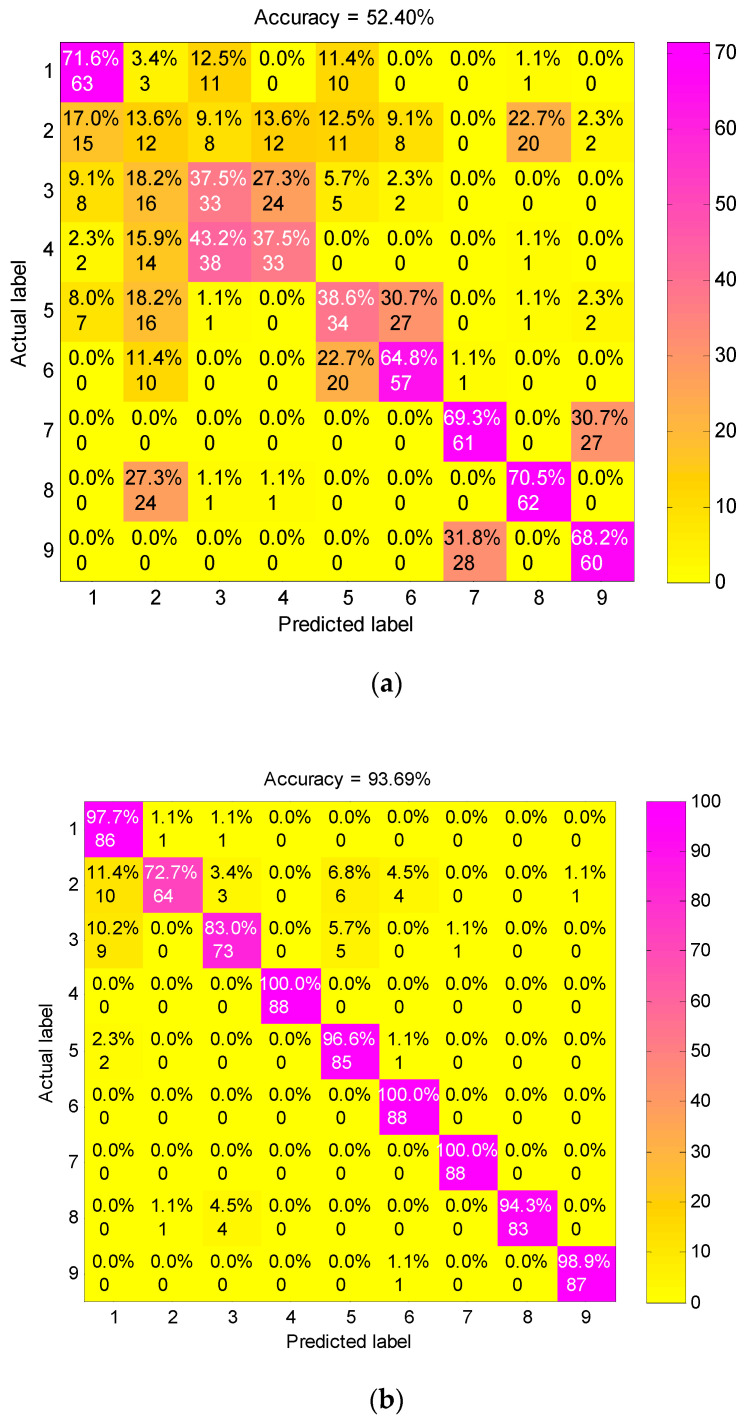

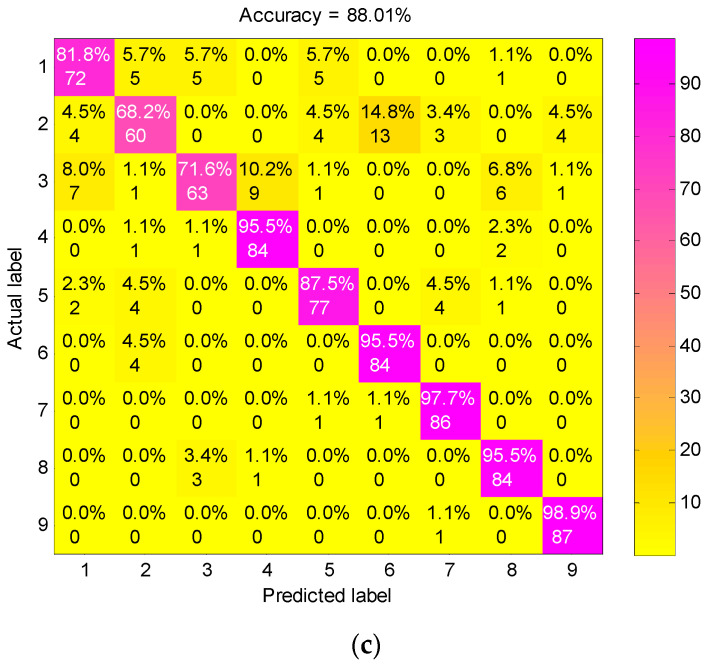
The confusion matrix of different feature extraction methods: (**a**) DE acting on raw vibration signals; (**b**) RCMDE acting on raw vibration signals; (**c**) IMF-DE method.

To validate the superiority of preprocessing the vibration signals by the presented method, we mainly compared the diagnosis performance of FEEMD-RCMDE with the following three feature extracting methods: single-scale DE acting on raw vibration signals, RCMDE acting on raw vibration signals and the method based on IMFs and DE (IMF-DE). The IMF-DE method is to calculate the DE of each IMF decomposed by FEEMD, the details of which can be found in the literature [31]. The features extracted by the above methods are presented into the RF classifier to identify all nine fault conditions. The proportion of training and test samples are the same as FEEMD-RCMDE, and the other conditions remain consistent. The confusion matrixes of different feature extraction methods for one trial are displayed in Figure 10. The classification accuracy of three methods DE, RCMDE and IMF-DE are 52.40%, 93.69%, 88.01%, respectively, which is far lower than the presented approach in Figure 8. The explanation for this is that DE is just a single-scale method. It can distinguish between normal and fault states under a certain scale factor as shown in Figure 4, but it struggles to identify different types of faults. RCMDE is a multi-scale method based on raw data, which can achieve a better classification result than the single-scale method. IMF-DE is an entropy-based method combined with the time–frequency analysis technique, but it merely measures the complexity of signals from a single scale. Compared with the above method, the generalized multi-scale feature extraction method FEEMD-RCMDE is a multi-scale entropy-based method combined with the time–frequency technique. It can reflect more fault information hidden in the raw vibration signals via different frequency components and different scales, which can better distinguish faults of different types and severities.

**Figure 11:**
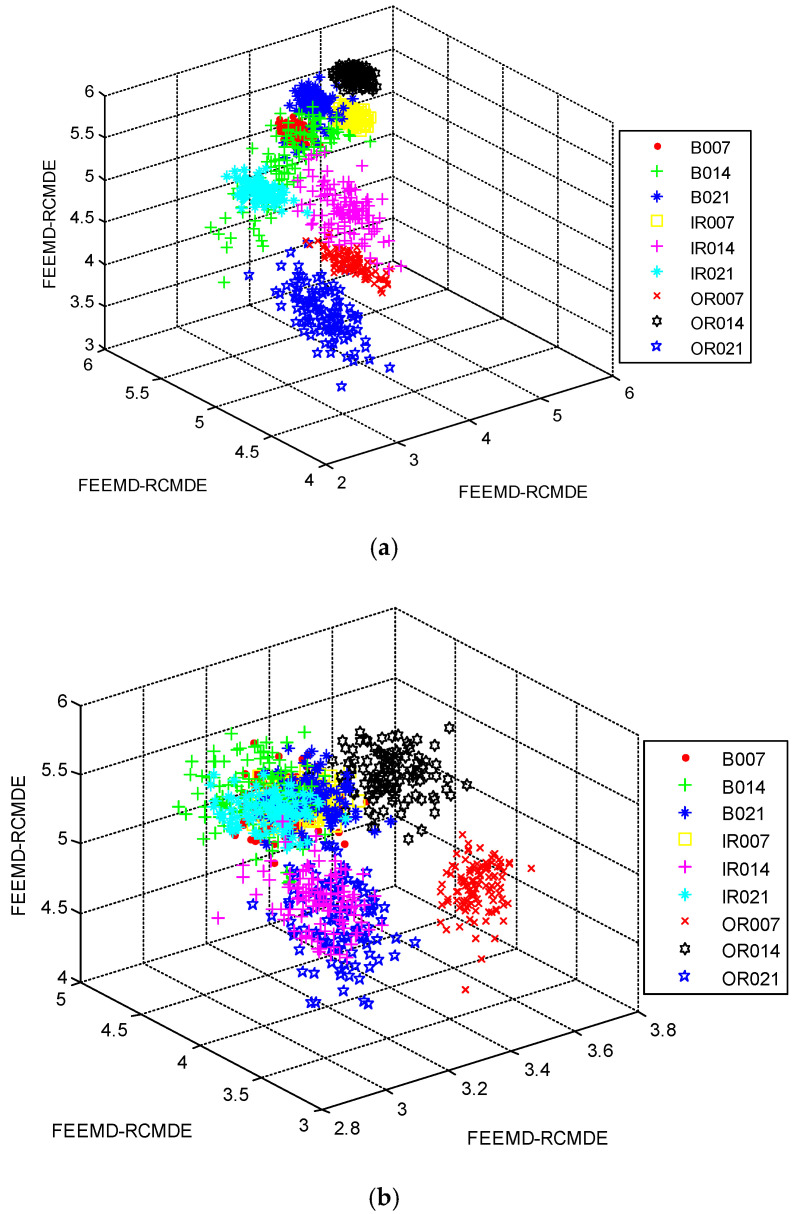
Three-dimensional view of the first three features by FEEMD-RCMDE: (**a**) Using mRMR; (**b**) without using mRMR.

**Figure 12:**
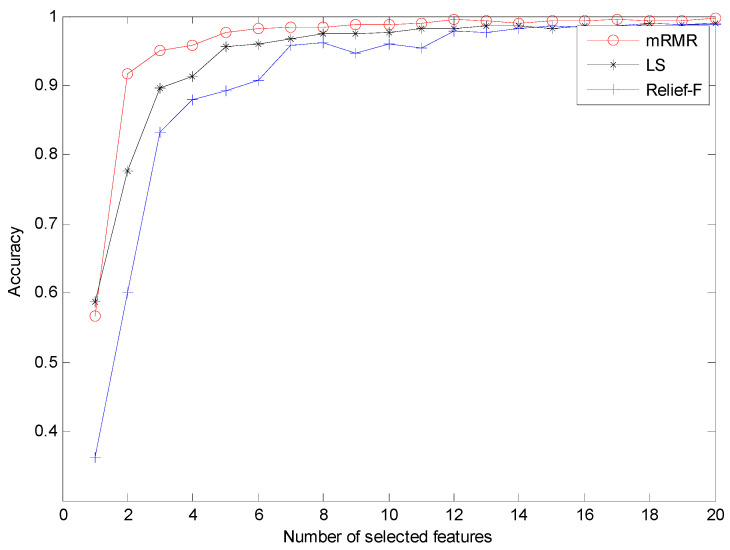
The diagnosis results of different feature selection methods. LS = Laplacian score.

In order to investigate the necessity of the mRMR approach, we randomly selected 12 features of different methods to train the RF classifier for fault diagnosis. The classification accuracies of different methods are displayed in Table 3. Evidently, FEEMD-RCMDE achieves the best classification results, and the classification accuracies of different methods without mRMR are lower than that in Table 2. The three-dimensional view of three FEEMD-RCMDE features for all fault working conditions are presented in Figure 11. Figure 11a shows the selected features using mRMR, while Figure 11b shows the random selected features. The result indicates that different fault working conditions are better separated in Figure 11a compared with Figure 11b. Meanwhile, it can be found that some samples of different fault working conditions overlap in Figure 11a, such as B007 and B014. The findings agree with the result of misclassification in Figure 7. The reason is that it is not enough to distinguish all fault working conditions by only three selected features. Thus, to further study the advantages of the mRMR approach and explore the optimal number of feather two typical feature selection methods, Laplacian score (LS) [45] and Relief-F [46], are also performed for comparison. LS is a widely applied unsupervised learning approach, in which the importance of a feature is assessed by scoring the ability of locality preserving. Relief-F is a supervised approach that judges the importance of a feature by the value of neighborhood data samples. The diagnosis results of three feature selection methods are presented in Figure 12. As Figure 12 shows, the classification result by the mRMR approach is better than the other two feature selection approaches. Moreover, as the number of selected features increases, the classification accuracy becomes higher. When it reaches 12, the accuracy by the mRMR approach achieves over 99%. It will not contribute too much to classification accuracy by increasing the number of features, but will waste the computational cost. Therefore, considering the efficiency and effectiveness, the number of selected features in this study is set to 12. 

To analyze the influence of different classifiers on the diagnosis results, the features by the proposed method were sent into support vector machine (SVM) and extreme learning machine (ELM) classifiers for comparison. The output classification results and average CPU time are listed in Table 4. As Table 4 shows, the SVM classifier achieved the best classification results, but its model parameters are difficult to determine and the CPU time is significantly larger than the other two methods. The RF classifier achieved a relatively high accuracy, which is just slightly lower than SVM, and its CPU time is also much lower. Taking into account efficiency and effectiveness, the RF classifier was presented for fault diagnosis in this study.

## 5. Conclusions

In this study, a comprehensive fault diagnosis approach is proposed to detect faults and identify different fault working conditions of bearings. In the progress of fault detection, the distribution of RCMDE values with the change of scale factor under different working conditions is investigated, and thus a threshold is defined which can effectively judge the health state of bearings. If the fault was detected, the generalized multi-scale feature extraction method FEEMD-RCMDE was employed to extract fault features to generate the candidate feature pool. Then, the sensitive features were selected by mRMR and presented into an RF classifier for pattern classification. The comparison results of the experiment among RCMDE, IMF-DE and FEEMD-RCMDE indicate that the proposed method can fully extract the fault information of vibration signals. Meanwhile, compared with other widely used entropy-based method such as MPE, MSE and MDE, the experimental results validate the superiority and effectiveness of the proposed method. Moreover, if the bearings work normally, the existence of fault detection can avoid unnecessary implementation of pattern classification. Thus, it can improve the efficiency and effectiveness of fault diagnosis, which is more appropriate for practical applications. 

## Figures and Tables

**Table 1:** The details of the experimental data.

Working Conditions	Severity (Inches)	Abbreviation	Number of Training Samples (20%)	Number of Testing Samples (80%)	Classification Label
Normal	None	N	22	88	0
Ball fault	0.007	B007	22	88	1
	0.014	B014	22	88	2
	0.021	B021	22	88	3
Inner race fault	0.007	IR007	22	88	4
	0.014	IR014	22	88	5
	0.021	IR021	22	88	6
Outer race fault	0.007	OR007	22	88	7
	0.014	OR014	22	88	8
	0.021	OR021	22	88	9

**Table 2:** The classification accuracies of different methods.

Different Methods	Accuracy (%)		
	Max	Min	Mean
The proposed method	100	98.36	99.27
FEEMD-MDE and mRMR	98.99	96.72	97.93
FEEMD-MPE and mRMR	97.10	94.44	95.69
FEEMD-MSE and mRMR	98.23	93.42	96.43

**Table 3:** The classification accuracies of different methods without mRMR.

Different Methods	Accuracy (%)		
	Max	Min	Mean
FEEMD-RCMDE	95.08	91.16	93.41
FEEMD-MDE	91.16	87.37	89.58
FEEMD-MPE	85.28	79.49	82.27
FEEMD-MSE	82.95	78.79	80.81

**Table 4:** The classification accuracies of the proposed method with different classifiers for 20 trials.

Different Classifier	Accuracy (%)			CPU Time (s)
	Max	Min	Mean	
RF	100	98.36	99.27	0.29
ELM	99.31	96.54	97.96	0.11
SVM	100	98.75	99.42	12.90
